# Distant parenchymal recurrence during long-term use of TTFields treatment for glioblastoma

**DOI:** 10.1007/s10147-025-02775-5

**Published:** 2025-05-22

**Authors:** Yuhei Takido, Fumiharu Ohka, Shoichi Deguchi, Kazuya Motomura, Koichi Mitsuya, Kosuke Aoki, Yoshiki Shiba, Kazuhito Takeuchi, Yuichi Nagata, Junya Yamaguchi, Yuji Kibe, Yutaro Fuse, Sachi Maeda, Hiroki Shimizu, Ryuta Saito

**Affiliations:** 1 Department of Neurosurgery, Nagoya University Graduate School of Medicine, 65 Tsurumai-Cho, Showa-Ku, Nagoya, 466-8550 Japan; 2 Division of Neurosurgery, Shizuoka Cancer Center, Nagaizumi, Japan

**Keywords:** TTFields treatment, Glioblastoma, Distant recurrence

## Abstract

**Background:**

Tumor treating fields (TTFields) treatment has been an important option for the treatment of glioblastoma. The introduction of novel treatment options may lead to distinct recurrence patterns compared to those observed with conventional therapies; however, the specific recurrence pattern during TTFields treatment has not been elucidated.

**Methods and results:**

Here, we analyzed 39 cases of glioblastoma treated with TTFields. Although a usage rate of more than 75% is recommended, among 39 cases, 18 discontinued TTFields treatment owing to requests by patients with lower usage rates. In these discontinued cases, patients exhibiting sensory aphasia were more frequently included compared to those who continued TTFields (44.4%,* p* < 0.001). Among 21 cases involving patients who continued TTFields, tumor recurrence was observed in 15 of those cases. Five out of 15 cases (33.3%) exhibited recurrence in distant parenchyma from the primary lesion. A higher usage rate and relatively longer use of TTFields were observed in these five cases, along with more favorable progression-free survival than those in the other 10 cases (*p* = 0.019,* p* = 0.040, and *p* = 0.024, respectively). In one case, recurrent tumors with lower grade glioma histology but molecular markers characteristic for glioblastoma, IDH-wildtype were indentified. This tumor arose in an area that received a lower local minimum power density of TTFields compared to the primary lesion, following long-term TTFields therapy.

**Conclusions:**

Long-term use of TTFields might be correlated with a high frequency of distant parenchymal recurrence in cases with favorable response.

**Supplementary Information:**

The online version contains supplementary material available at 10.1007/s10147-025-02775-5.

## Introduction

Glioblastoma is one of the most lethal central nervous system (CNS) tumors. A recent comprehensive molecular analysis of this type of glioma revealed various important gene alterations [1–3], introducing a large number of clinical trials targeting specific gene alterations. However, a novel potent systemic chemotherapy reagent has not been identified yet, since the identification of temozolomide (TMZ) and bevacizumab (BEV). In 2015, Tumor treating fields (TTFields) therapy was approved by the FDA for the treatment of newly diagnosed glioblastoma cases. TTFields delivered low-intensity, intermediate-frequency, 200 kHz alternating electric fields, inhibiting tumor cell proliferation by interfering with mitotic spindle formation during metaphase [4, 5]. During the EF-14 randomized trial, TTFields combined with TMZ significantly improved progression-free survival (PFS) and overall survival (OS) compared with only TMZ treatment (PFS; 6.7 months vs 4 months and OS; 20.9 months vs 16.0 months in TTFields with TMZ group or just TMZ group, respectively) [6, 7]. Based on these results, TTFields treatment has been a widely used treatment option for newly diagnosed glioblastoma. In post hoc analysis of EF-14, a favorable prognosis was recorded in cases where the usage rate of TTFields device was more than 75% (average of 18 h in a day) compared to those with less than 75% usage rate [7]. Therefore, encouraging high and long-term use is important to improve prognosis during the treatment of TTFields.

Diffuse high-grade glioma exhibits highly infiltrative biology, resulting in recurrence after initial treatment using multimodality. During the treatment of diffuse high-grade glioma, immediate identification of tumor recurrence is essential. To date, among glioblastoma cases treated by tumor removal followed by radiation therapy with concurrent and adjuvant TMZ, local recurrence around the primary tumor lesion was recorded in 87.5% of these cases, while the frequency of distant or multifocal recurrence was quite low [8]. However, the recurrence pattern of glioblastoma treated with TTFields has not been elucidated.

In this study, we retrospectively analyzed 39 cases treated with TTFields. Among 39 cases, in 18 cases, TTFields treatment was discontinued owing to requests by patients except for tumor progression. In these cases, those with sensory aphasia symptoms before the introduction of TTFields were detected more frequently than those who continued TTFields treatment. Among continued cases, recurrence in brain parenchyma distant from the primary lesion was observed in five cases. These five cases exhibited distant recurrence after long-term use of TTFields treatment with favorable PFS compared with the other cases. These findings showed that for cases with favorable responses for TTFields, we need to care for distant parenchymal recurrence during the clinical course.

## Materials and methods

### Etiology

This study was approved by the Institutional Review Board of Nagoya University Hospital (approval number: 2021-0451 and 2023-0319) and complied with all provisions of the World Medical Association Declaration of Helsinki.

### Patient data

We encountered glioblastomas in adult patients who started TTFields treatment between April 2018 and May 2023 at Nagoya University Hospital (Nagoya, Japan) and Shizuoka Cancer Center (Nagaizumi, Japan). Patient data on clinical information and outcomes, including age, sex, past medical history, family history, histopathological findings, extent of resection, prescribed adjuvant therapy, radiographic findings before and after treatment, average usage rate of TTFields treatment, days of TTFields treatment, PFS and OS were retrospectively analyzed. Age and KPS were evaluated at the time of TTFields induction. Tumor volume/location and pre-operative presentation were evaluated with pre-operative MRI of the first surgery. Extent of resection was evaluated with pre- and post-operative MRI of the first surgery. PFS and OS were defined based on the duration from introduction of TTFields treatment to recurrence.

### DNA extraction from tumor and blood samples

Tumor samples were obtained intraoperatively. DNA was extracted from frozen tumors and blood samples using a QIAamp DNA Mini Kit (Qiagen, Hilden, Germany) following the instructions of the manufacturer. We extracted DNA from formalin-fixed paraffin-embedded (FFPE) samples of recurrent tumors using the GeneRead DNA FFPE Kit (Qiagen) following the instructions of the manufacturer. The amount of DNA obtained was evaluated using a Qubit dsDNA HS Assay Kit (Invitrogen, Paisley, Scotland).

### Sanger sequencing and multiplex ligation-dependent probe amplification (MLPA)

Sanger sequencing was performed to detect mutations in *IDH1, IDH2*, and *TERT* promoter. We amplified a 129-, 239-, or 237-base pair fragment for the sequence encoding R132 of the *IDH1* gene, R172 of the *IDH2* gene, or C228/ C250 of the *TERT* promoter, respectively. For *IDH1* and *IDH2* sequencing, we applied conventional PCR following these steps: 35 cycles of denaturation at 98 °C for 10 s, annealing at 62 °C for 30 s, and extension at 68 °C for 30 s, with a final extension step at 68 °C for 5 min. *IDH1* forward primer (5′-CGGTCTTCAGAGAAGCCATT-3′) and reverse primer (5′-GCAAAATCACATTATTGCCAAC-3′), and *IDH2* forward primer (5′- TGTGGAAAAG-TCCCAATGGA-3′) and reverse primer (5′-TGTGGCCTTGTACTGCAGAG-3′) were used. For *pTERT* sequencing, we applied a conventional PCR following these steps: 35 cycles of denaturation at 98 °C for 10 s, annealing at 65 °C for 30 s, and extension at 68 °C for 1 min, with a final extension step at 68 °C for 5 min using the forward primer (5′-CGGTCTTCAGAGAAGCCATT-3′) and reverse primer (5′-GCAAAATCACATTATTGCCAAC-3’). Sequencing analysis was performed using the ApE software (v2.0.70). MLPA was used to detect 1p/19q codeletion, + 7/–10, and *EGFR* amplification [9]. The analysis was performed using the SALSA MLPA KIT P088-C1, D1, and P105-D1, D2, and D3, in accordance with the protocol of the manufacturer (MRC Holland, Amsterdam, Netherlands) [10]. Amplification products were separated using an ABI 3730 Genetic Analyzer (Applied Biosystems, Foster City, CA, USA). Using the Coffalyser.Net software, data analysis, including quantification, was performed following the MRC-Holland procedures.

### Statistical analysis

All statistical analysis was performed using IBM SPSS version 28.0 (IBM Corp.). Chi-square or Fisher’s exact tests were used when appropriate to compare categorical variables. Age, Karnofsky Performance Status (KPS) scores, tumor volume, extent of resection, average usage rate of TTFields treatment, and days of TTFields treatment were compared between groups using the Mann–Whitney *U*-test. The PFS and OS of patients with and without distant parenchymal recurrence were plotted using the Kaplan–Meier method and log-rank analysis was used to compare the plots.

## Results

### Clinical characteristics of the analyzed cases

The clinical characteristics of the 39 cases analyzed, including sex and age, are described in Table 1. In all the cases, pathologic diagnosis or integrative diagnosis with pathologic and molecular analysis of the tumor specimens obtained by initial biopsy or tumor removal was glioblastoma according to the World Health Organization (WHO) classification revised 4th edition [11] or glioblastoma, IDH-wildtype according to the WHO classification 5th edition [1]. A total of 20 and 19 cases treated at the Nagoya University Hospital and Shizuoka Cancer Center, respectively, were included. The pre-operative symptoms were hemiparesis (*n* = 12), seizure (*n* = 10), sensory aphasia (*n* = 9), headache (*n* = 5), and motor aphasia (*n* = 4). The primary tumor locations were the frontal (*n* = 17), temporal (*n* = 10), and parietal (*n* = 4) lobes. The median tumor volume was 27.32 mL (11.77–44.55 mL). Initial surgery was performed as follows: biopsy (*n* = 4), gross total resection (GTR; *n* = 22), subtotal resection (STR; *n* = 9), and partial resection (PR; *n* = 4).

**Table 1:** Overview of patient and tumor characteristic

Characteristic	Value
No. of patients	39
Sex	
Male	22 (56.4%)
Female	17 (43.6%)
Age, years	54 (40–67)
Preop symptom	
Hemiparesis	12 (30.8%)
Motor aphasia	4 (10.3%)
Sensory aphasia	9 (23.1%)
Seizure	10 (25.6%)
Headache	5 (12.8%)
Cognitive decline	3 (7.7%)
Visual field defect	2 (5.1%)
Gerstmann syndrome	1 (2.6%)
Psychomotor agitation	1 (2.6%)
MRI (incidental)	1 (2.6%)
Tumor location	
Frontal	17 (43.6%)
Frontal-basal ganglia	1 (2.6%)
Parietal	4 (10.3%)
Parietooccipital	1 (2.6%)
Temporoparietal	3 (7.7%)
Temporal	10 (25.6%)
Temporal-insular	1 (2.6%)
Temporal-thalamus	1 (2.6%)
Thalamus	1 (2.6%)
Operation type	
Craniotomy	35 (89.7%)
Endoscopic surgery	4 (10.3%)
Tumor volume, ml	27.32 (11.77–44.55)
Extent of resection	
Biopsy (0–50%)	4 (10.3%)
PR (50–80%)	4 (10.3%)
STR (80–98%)	9 (23.1%)
GTR (98%-)	22 (56.4%)
IDH1 R132H mutation	
Positive	3 (7.7%)
Negative	36 (92.3%)
KPS score	80 (70–90)

Values are presented as number (%) of patients or median (IQR)

*PR* partial resection, *STR* subtotal resection, *GTR* gross total resection

### Usage rate and length of TTFields treatment

Among the 39 cases, TTFields treatment was discontinued in 18 cases as requested by the patients (discontinued group), while TTFields treatment continued in 21 cases until progression or completion, or have been continuing TTFields treatment (continued group). Patient-requested discontinuations included only the patient who did not attempt to complete the recommended TTFields treatment and discontinued at the patient's request even without tumor recurrence. The clinical characteristics of these cases are described in Table 2. Patients in the continued group underwent TTFields treatment for longer periods than those in the discontinued group (median 385 days (241–487 days) and 42 days (23–152 days), respectively; *p* < 0.001). Also, patients in the continued group showed significantly higher usage rates than those in the discontinued group (median 84% (65–90%) and 49% (0–79%), respectively;* p* < 0.001). Comparing various clinical findings between these subgroups, older cases were more frequently found in the discontinued group (*p* = 0.019). Also, pre-operative mild or severe sensory aphasia symptoms were observed more frequently in the discontinued group than in the continued group (eight cases in the discontinued group and no case in the continued group, *p* < 0.001). Temporal lobe tumor in the dominant hemisphere was more frequently observed in the discontinued group than in the continued group, although this observation was not statistically significant. (*p* = 0.153). The extent of resection and KPS score was not significantly different between these groups. These findings suggested that sensory aphasia symptom is correlated with low usage rate and discontinuation as requested by the patients together with older age. Median PFS by the Kaplan–Meier method was 336 days in the continued group and 290 days in the discontinued group (*p* = 0.653), and median OS was 632 days and 593 days, respectively (*p* = 0.552). There was no statistically significant difference in PFS/OS between the continued and discontinued groups, possibly due to the short observation period and small number of cases. However, identification of the factors leading to discontinuation and low usage rates is important to help derive maximum benefit from the efficacy of TTFields demonstrated in the previous reports.

**Table 2:** Usage condition of TTFields

Characteristic	Continued group	Discontinued group	*p* value
No. of patients	21	18	
Sex			0.232
Male	10 (47.6%)	12 (66.7%)	
Female	11 (52.4%)	6 (33.3%)	
Age, years	51 (37–59)	62.5 (53–70.5)	**0.019**
Preop presentation (moderate or severe sensory aphasia)	0 (0%)	8 (44.4%)	** < 0.001**
Tumor location (Temporal lobe of the dominant hemisphere)	3 (14.3%)	6 (33.3%)	0.153
Tumor volume, ml	23.40 (12.24–40.92)	28.92 (10.80–58.36)	0.379
Extent of resection			0.900
Biopsy (0–50%)	3 (14.3%)	1 (5.6%)	
PR (50–80%)	0 (0%)	4 (22.2%)	
STR (80–98%)	6 (28.6%)	3 (16.7%)	
GTR (98%-)	12 (57.1%)	10 (55.6%)	
IDH1 R132H mutation			0.146
Positive	3 (14.3%)	0 (0%)	
Negative	18 (85.7%)	18 (100%)	
KPS score	90 (70–90)	80 (70–90)	0.922
Average usage rate of TTFields treatment (%)	84 (65–90)	49 (0–79)	** < 0.001**
Days of TTFields treatment	385 (241–487)	42 (23–152)	** < 0.001**

Values are presented as number (%) of patients or median (IQR). Boldface type indicates statistical significance

### Tumor recurrence in the continued group

**Fig. 1:**
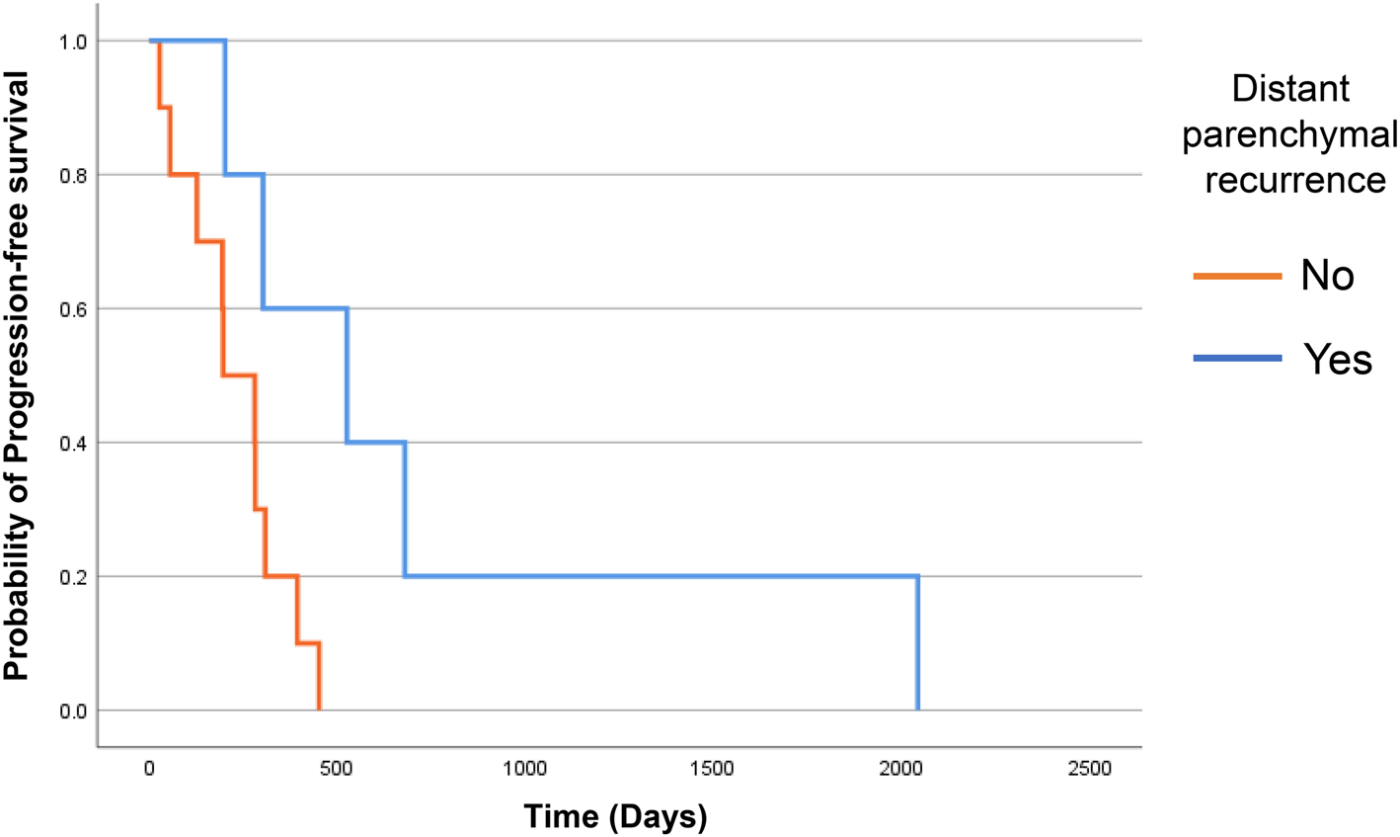
Kaplan–Meier curve indicating PFS. Kaplan–Meier curve indicating PFS of patients with distant parenchymal recurrence (*n* = 5; blue line) and patients without distant parenchymal recurrence (*n* = 10; orange line; *p* = 0.024)

Among 21 cases in the continued group, recurrence during TTFields treatment was observed in 15 cases. Of these 15 cases, 10 cases exhibited local recurrence around the primary tumor and one of the 10 cases revealed additional leptomeningeal dissemination. By contrast, in five cases, patients exhibited recurrence in the distant parenchyma from the primary lesion. Compared with data obtained from a previous study which reported the recurrence pattern of glioblastoma treated without TTFields treatment, distant parenchymal recurrence was more frequently observed in these 15 cases (*p* < 0.01) [12, 13]. Among the five cases who exhibited recurrence in the distant parenchymal from the primary lesion, one, two, and two cases exhibited recurrence in another lobe of the ipsilateral hemisphere, in the contralateral hemisphere, and the cerebellum, respectively. In these five cases, higher usage rates and long-term use were recorded compared to the 10 cases (median 88% (73–89%) vs 65% (51–75%) and median 525 days (252–1361 days) vs 238 days (108–329 days),* p* = 0.019 and *p* = 0.040, respectively). Also, more favorable PFS were observed in these cases compared to other cases (*p* = 0.024; Fig. 1). Median PFS by the Kaplan–Meier method was 525 days in the group of distant parenchymal recurrence and 196 days in the group of non-distant parenchymal recurrence, and median OS was 679 days and 548 days, respectively (*p* = 0.141; *supplementary* Fig. 1). There was a trend toward longer OS in the distant parenchymal group, although there was no statistically significant difference in OS due to the short observation period and small number of cases. These findings suggested that atypical recurrence such as those in distant parenchyma may be observed in cases where TTFields treatment is used for long periods.

### Two characteristic cases exhibiting recurrence in distant parenchyma after long-term TTFields treatment

#### Case 1

**Fig. 2:**
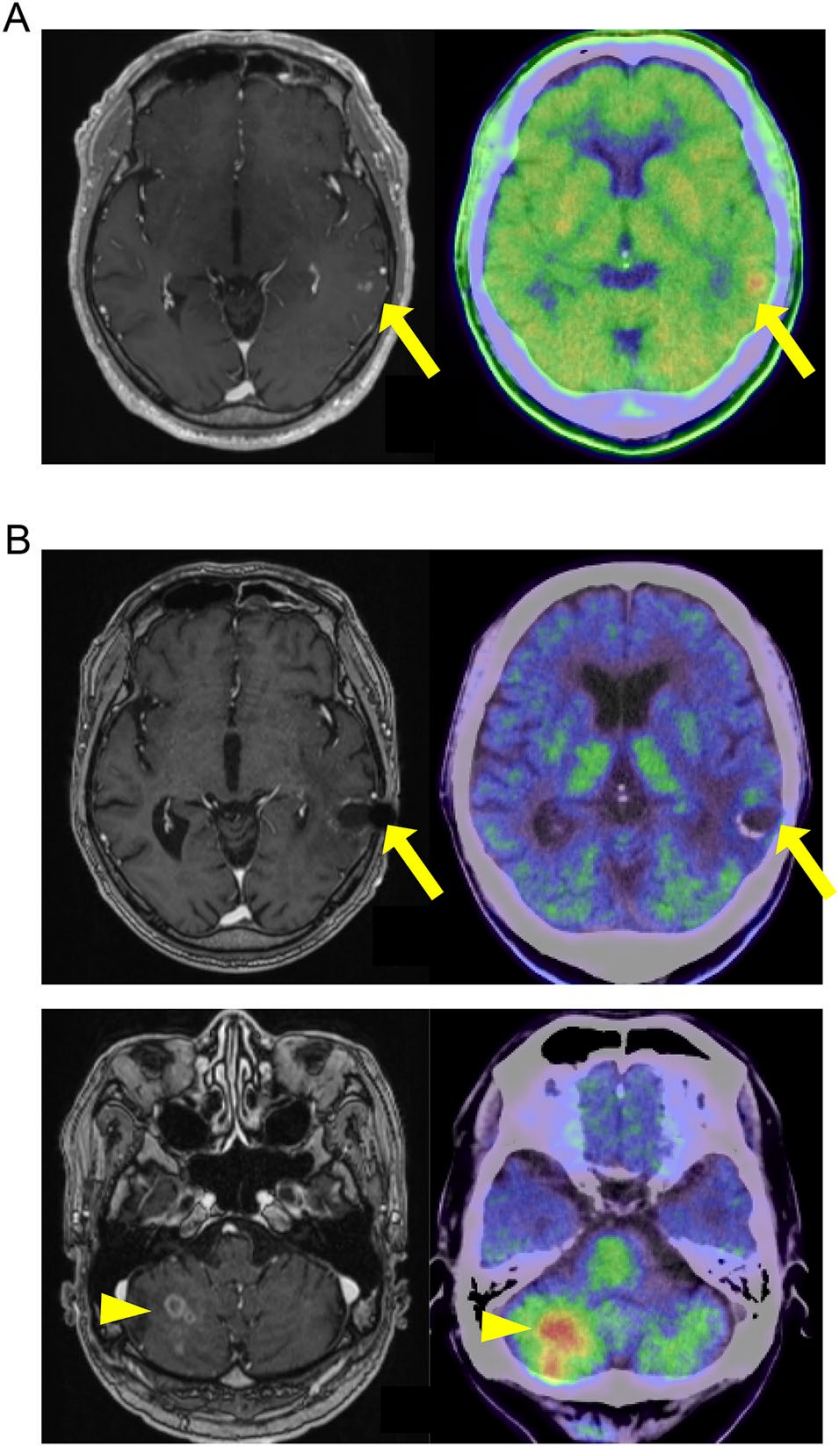
MRI and Methionine-PET images of Case 1. (**A**) Pre-operative MRI contrast-enhanced T1-weighted image (left) and Methionine-PET (right). Yellow arrow indicates tumor lesion. (**B**) MRI contrast-enhanced T1-weighted image after radiation therapy concomitant with TMZ. Yellow arrow indicates tumor lesion. (**C**) MRI contrast-enhanced T1-weighted image (left) and Methionine-PET (right) of the primary lesion (upper) and cerebellum (lower) on recurrence. Yellow arrows and arrow heads indicate primary lesions and recurrent lesions, respectively

In a 71-year-old male, an MRI revealed a small gadolinium-enhanced lesion in the left temporal lobe (Fig. 2A). Biopsy analysis revealed glioblastoma, IDH-wildtype, based on both histologic and molecular evaluation of the tumor tissue. Immediately after the biopsy, he underwent radiation therapy (60 Gy/30 fr) concomitant with TMZ. After that, he started maintenance therapy using TTFields treatment with TMZ (Fig. 2B). He could maintain stable disease (SD) status continuing TTFields treatment with TMZ. However, after 17 months, an MRI revealed a recurrence of a gadolinium-enhanced lesion in the right cerebellum hemisphere, while the primary lesion maintained SD status (Fig. 2C). BEV treatment was introduced; however, 25 months after the initial biopsy, the patient died owing to progression of the tumor (Table 3).

**Table 3:** Recurrence during TTFields treatment

Characteristic	Distant parenchymal recurrence	No distant parenchymal recurrence	*p* value
No. of patients	5	10	
Sex			0.287
Male	2 (40.0%)	7 (70.0%)	
Female	3 (60.0%)	3 (30.0%)	
Age, years	42 (29–74)	52 (35.25–62.50)	1.000
Local recurrence around primary lesion	2 (40.0%)	10 (100%)	**0.022**
Leptomeningeal metastases	0 (0%)	1 (9.1%)	0.667
Distant parenchymal recurrence	5 (100%)	0 (0%)	
Another lobe of the ipsilateral hemisphere	1 (20.0%)	–	
Contralateral hemisphere	2 (40.0%)	–	
Cerebellum	2 (40.0%)	–	
Tumor volume, ml	25.42 (1.16–52.35)	25.36 (15.63–39.50)	0.768
Extent of resection			0.075
Biopsy (0–50%)	0 (0%)	3 (30.0%)	
PR (50–80%)	0 (0%)	1 (10.0%)	
STR (80–98%)	1 (20.0%)	3 (30.0%)	
GTR (98%-)	4 (80.0%)	3 (30.0%)	
IDH1 R132H mutation			0.429
Positive	0 (0%)	2 (20.0%)	
Negative	5 (100%)	8 (80.0%)	
KPS score	80 (70–85)	85 (70–90)	0.594
Average usage rate of TTFields treatment (%)	88 (73–89)	65 (51–75)	**0.019**
Days of TTFields treatment until first recurrence	525 (252–1361)	238 (108–329)	**0.040**

Values are presented as number (%) of patients or median (IQR). Boldface type indicates statistical significance

#### Case 2

**Fig. 3:**
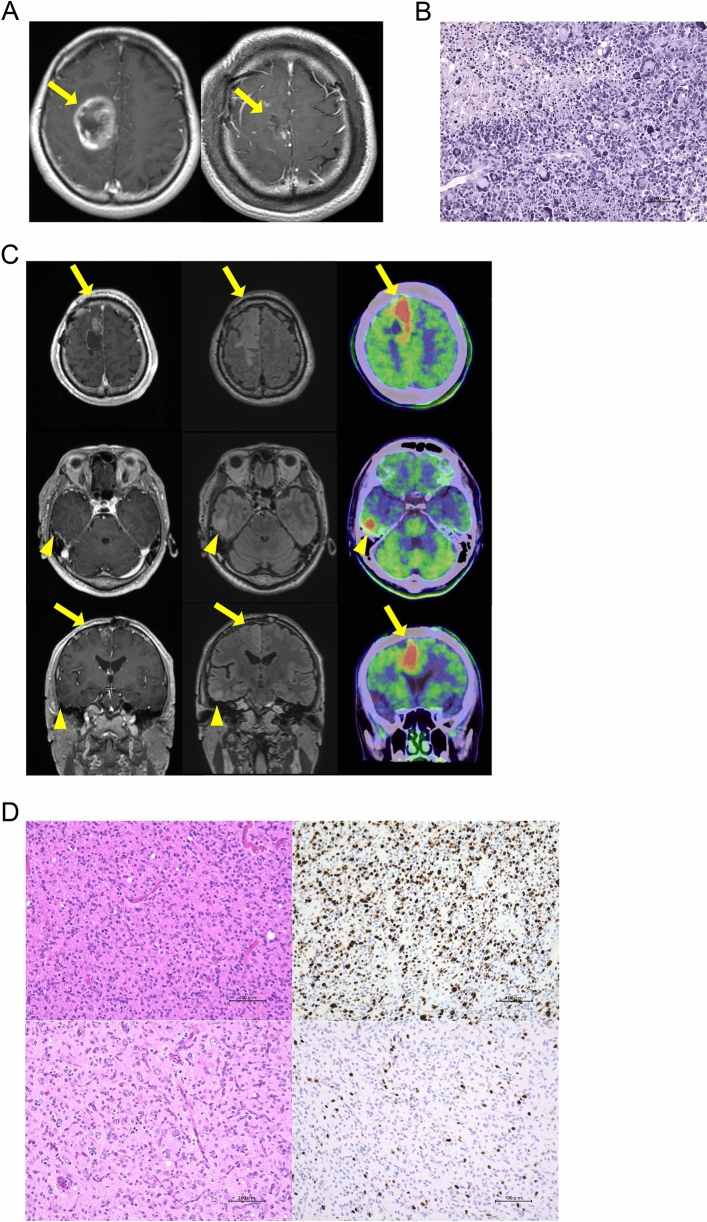
MRI, Methionine-PET, and Hematoxylin and Eosin (HE) images of Case 2. (**A**) Pre-operative (left) and post-operative (right) MRI contrast-enhanced T1-weighted image. Yellow arrows indicate tumor lesion. (**B**) HE image of primary tumor. A scale bar indicates 100 um. (**C**) MRI contrast-enhanced T1-weighted image (left), FLAIR images (middle), and Methionine-PET (right) of the recurrent lesion. Axial image (upper and middle) and coronal image (lower). Yellow arrows and arrow heads indicate recurrent tumors around primary lesions and on distant parenchyma, respectively. (**D**) HE image (left) and IHC using anti-Ki67 antibody (right) of recurrent tumor around the primary lesion (upper) and on distal parenchyma (lower). Scale bars indicate 100 um

**Fig. 4:**
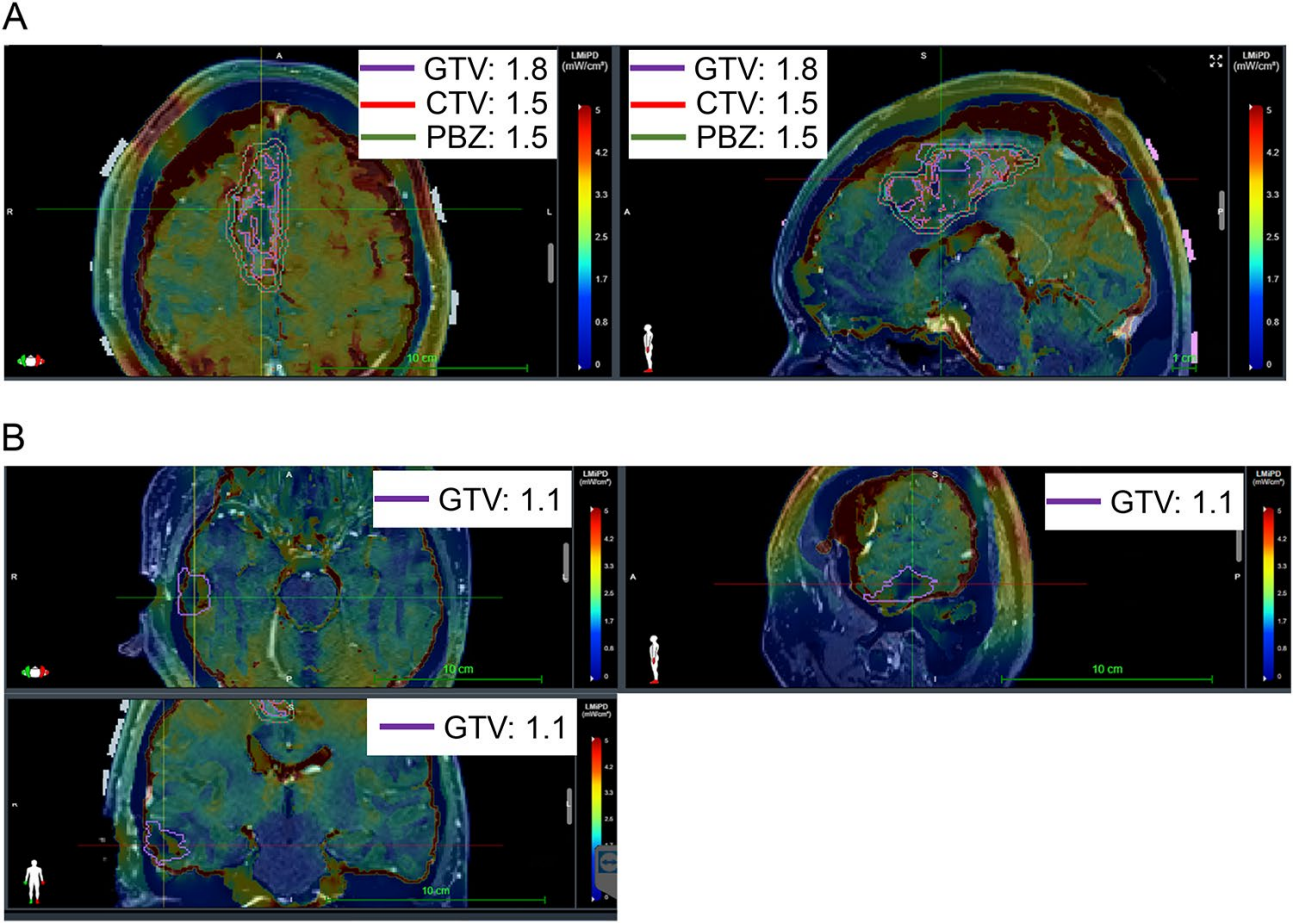
Electric fields map for recurrent lesions of Case 2. (**A**) Axial (left) and sagittal (right) images of electric fields for a recurrent tumor on the primary lesion. The purple, red or green line indicates gross tumor volume (GTV; 1.8 LMiPD), clinical target volume (CTV; 1.5 LMiPD) and peritumoral boundary zone (PBZ; 1.5 LMiPD), respectively. (**B**) Axial (left, upper), sagittal (right, upper), and coronal (left, lower) image of electric fields for recurrent tumor on distant parenchyma. The purple line indicates GTV (1.1 LMiPD) of a recurrent lesion

A 42-year-old male developed left hemiparesis and partial seizure. MRI revealed a gadolinium-enhanced mass lesion in the right frontal lobe. GTR was achieved and the integrative diagnosis was glioblastoma, IDH-wildtype (Fig. 3A, B). Immediately, he underwent radiation therapy (60 Gy/30 fr) concomitant with TMZ. After that, he started maintenance therapy using TTFields treatment with TMZ. For 67 months, he could maintain complete response (CR) status by continuing TTFields treatment with TMZ. MRI and Methionine-PET revealed recurrent tumors around the primary tumor lesion together with those in the right temporal lobe (Fig. 3C). Tumor removal for both lesions was performed. Pathologic findings of recurrent tumors around the primary lesion and temporal lobe were consistent with those of grade III glioma with 30% of Ki67 index and grade II glioma with 5–10% of Ki67 index according to the WHO classification revised 4th edition, respectively (Fig. 3D). Both tumors revealed *TERT* promoter mutation, *CDKN2A* homozygous deletion and *EGFR* amplification without IDH mutation. An electric fields map provided by Novocure revealed that the local minimum power density (LMiPD) of the TTFields was lower in the temporal lesion compared to those in the primary lesion (Fig. 4). Because temporal lesions were outside of area treated with prior radiation therapy, after tumor removal, we performed radiation therapy for temporal tumor as recurrent glioblastoma, IDH-wildtype. These cases suggested that during long-term use of TTFields therapy, not only local recurrence around primary lesion but distant recurrence can occur outside the area targeted by TTFields, such as in the inferior tentorial lesion and in areas with lower TTField exposure.

## Discussion

In this study, we analyzed 39 cases treated with TTFields. Among these cases, symptoms of sensory aphasia may be owing to the short use and discontinuation as requested by patients as well as low usage rate. In cases where TTFields treatment continued, tumor recurrence in distant parenchyma was more frequently observed than previously reported [14]. In one case who was treated with TTFields for 67 months, tumor recurrence with histological characteristics of lower grade glioma was observed, exhibiting *TERT* promoter mutation, *CDKN2A* homozygous deletion, and *EGFR* amplification without IDH mutation in lower TTFields LMiPD area along with local recurrence.

A study on EF-14 revealed that mild to moderate skin irritation owing to the transducer arrays of TTFields occurred in more than half of the cases during TTFields treatment [7]. In addition to skin irritation, it might be a burden to continuously carry a device on a shaved scalp, resulting in a low usage rate or discontinuation as requested by patients. In the EF-14 study, more than 75% of the usage rate for 3 months resulted in a favorable prognosis. Among 450 cases, 75% of usage rate was not achieved in 185 cases (41.1%). In our 39 cases, lower usage rates and discontinuation as requested patients were recorded in 18 cases (46.2%). In these cases, ones exhibiting sensory aphasia were more frequently found compared to those who continued with a high usage rate. TMZ treatment was also reported to be sometimes interrupted by psychiatric symptoms [15]. However, patients in all of our 18 cases could continue TMZ treatment. Therefore, sensory aphasia symptoms may be responsible for the interruption of TTFields treatment. To increase the usage rate for these cases, social or daily support from a caregiver might be important since introduction of TTFields treatment.

The recurrence pattern of glioblastoma consists of local, dissemination, and distant recurrence owing to infiltration along the white matter tracts. The most frequent pattern is local recurrence which is reported in 87.5% of recurrent cases treated with TMZ [8]. Another report revealed that local progression, dissemination, or distant progression were observed in 75.3%, 18.6%, or 6.1% of glioblastoma cases treated with chemoradiotherapy, respectively [12]. Dissemination spreading of tumors is another important progression pattern of glioblastoma. Dissemination recurrence is also a rare pattern, however, 13% of newly diagnosed glioblastoma revealed spinal dissemination at the onset [16]. A shift of the recurrence pattern of glioblastoma from local recurrence to distant recurrence by the introduction of adjuvant therapies such as TMZ and BEV has been suggested [17]. Although, the addition of TTFields treatment might also shift the recurrence pattern of glioblastoma, the recurrence pattern of TTFields was not elucidated. In our cases, five cases (33.3%) with long-term use of TTFields treatment among 15 recurrent cases during TTFields treatment exhibited recurrence in distant parenchyma. Among five patients exhibiting distant parenchymal recurrence, BEV was not introduced as an initial treatment before the first recurrence. Notably, among the 10 cases exhibiting local recurrence, BEV was introduced as an initial treatment protocol for 3 cases. There was no statistically significant difference in the use of BEV before first recurrence between the group with and without distant parenchymal recurrence (*p* = 0.264). Among three patients with recurrence, two patients had local recurrence with enhanced masses, and one patient had a diffuse recurrence of non-enhanced lesion around initial tumor cavity. Considering that these distant recurrences were detected more frequently than those reported previously, the effect of TTFields might be correlated with distant recurrence in areas out of TTFields or where the LMiPD of the TTFields was low. During TTFields treatment, in two (13.3%) among 15 recurrent cases, tumor recurrence occurred in the cerebellar hemisphere. Recurrence in the cerebellar hemisphere is quite rare [18]. Kawauchi et al. reported six cases (4.5%) among 134 glioblastoma, IDH-wildtype cases revealed recurrence in the cerebellar hemisphere. Among six cases, no case of a patient who underwent TTFields treatment was recorded [19]. Kanamori et al. reported eight (7.4%) among 108 high-grade glioma cases where recurrence in the cerebellar hemisphere was observed. Among the eight cases, one case was treated with TTFields. In these studies, distant recurrence owing to infiltration along the white matter tracts was suggested [20]. Based on the improvement of local control of tumors by progression of treatment, recurrence by infiltration along the white matter tracts was reported to increase [17]. During TTFields treatment, recurrence in areas out of the TTFields, such as the cerebellar hemisphere, might be more frequently detected compared to those previously reported, especially in favorable response cases. In addition, even in the supratentorial area where the LMiPD of TTFields is low, recurrence as histologic findings of lower grade glioma might occur. For long-term usage cases with favorable response, we need careful observation of not only areas around primary lesions but outside of TTFields or area where the LMiPD of TTFields is low. Especially, in area where the LMiPD of TTFields is low, recurrence tumor might exhibit MRI and pathologic findings of lower grade glioma in spite of typical molecular features of glioblastoma, IDH-wildtype due to modification of continuous TTFields treatment. After first recurrence, BEV was introduced for three of five patients in the group with distant parenchymal recurrence and for all seven patients in the group without distant parenchymal recurrence. We analyzed nine patients who continued BEV more than 4 weeks. Among these nine patients, six patients continued TTFields with BEV, and three did not. Three of six patients with TTFields and two of three patients without TTFields revealed a reduction of size of contrast enhancement area as the best response. Six patients, consisting of five among the six patients with BEV and TTFields and one among the three patients with only BEV, revealed progression or re-recurrence after introduction of BEV during the observation period. All patients revealed progression or re-recurrence around the initial tumor cavity. Progression or re-recurrence of both non-enhanced and enhanced lesions was found in five patients, while progression of just non-enhanced lesion, which might have resulted from the usage of BEV, was found in one patient treated with BEV and TTFields.

To obtain the maximum benefit from TTFields treatment, social support or support from caregivers is important, especially for patients exhibiting sensory aphasia. For long-term use cases with favorable responses to TTFields treatment, we need to carefully observe distant parenchymal recurrence.

## Supplementary Information

Below is the link to the electronic supplementary material.

Supplementary file1 (PPTX 69 KB)
