# Supplementary material for: Distant parenchymal recurrence during long-term use of TTFields treatment for glioblastoma
Source: Int J Clin Oncol. 2025 May 22;30(7):1309–18. doi: 10.1007/s10147-025-02775-5 (PMC12187799; doi:10.1007/s10147-025-02775-5)
Supplement: Supplementary file 1 — Supplementary file1 (PPTX 69 KB) [file 10147_2025_2775_MOESM1_ESM.pptx]

## Slide 1
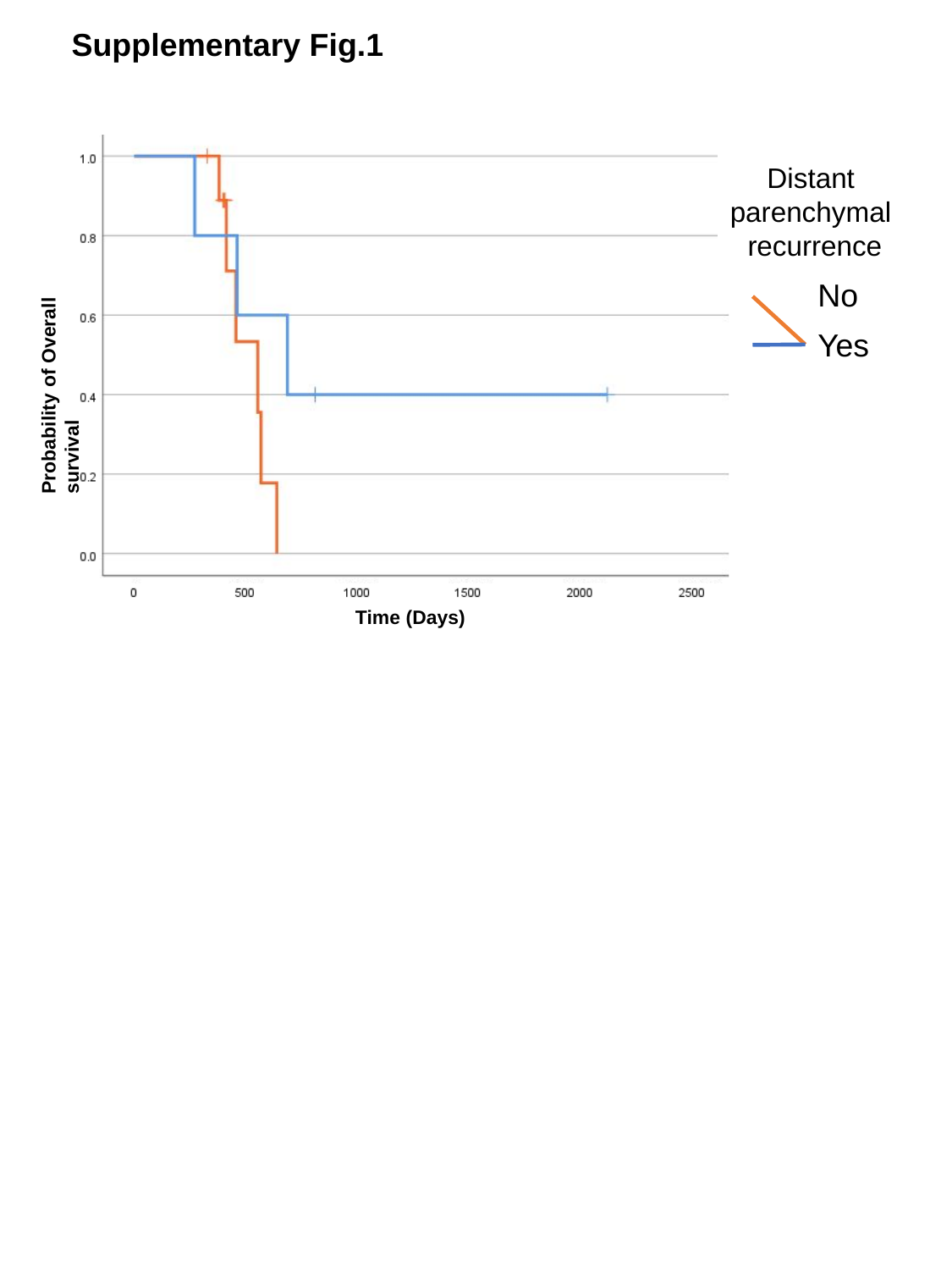

Supplementary Fig.1
Distant
parenchymal
recurrence
Probability of Overall survival
No
Yes
Time (Days)
